# The Prevailing Influenza

**Published:** 1848-03

**Authors:** 


### WESTMINSTER MEDICAL SOCIETY.

*The prevailing Influenza.*—Dr. Lankester, in allusion to the great prevalence of the present epidemic, observed that, contrasted with the influenza of former years, one of the characteristics of the present visitation was its greater prevalence and less severity. He had found among the patients attending the Pimlico Dispensary that many were effected by bronchitis; but out of one hundred and fifty instances of the disease, he had met with but two cases of pure pneumonia. Sore throat obtained in about half the cases; this symptom was attended by difficulty in swallowing. In some instances, the disease became even diptheritic. The general nervous symptoms, the depression and pain in the head, were similar to those which had been exhibited in former epidemics. The same law with respect to depletory measures held in this as in former visitations; for, however urgent the symptoms appeared, depletion was but ill borne. Generally, he thought the less we did the better, for debility would follow any kind of active treatment. He had seen one case assume all the characteristics of typhoid fever from too active treatment.

Dr. Ogier Ward said, that in Kensington the influenza presented the usual characteristics enumerated by Dr. Lankester. The pain in the head was the most general symptom; the sore throat prevailed in four-fifths of those attacked; and he had usually found it best relieved by an inhalation of warm vinegar and water. It was by no means a violent disease. The only case he had seen of much severity was a case similar to diptherite, and which he had successfully combated by Brettoneau's system. In some cases in which the pain in the head was most severe, the pain had been relieved by spontaneous bleeding from the nose. This might be a useful hint for practice, but he had never ventured on even local bloodletting. He then read a paper on

*The treatment of Asiatic cholera in the stage of collapse.*—He confined his observations to the collapsed stage as being most fatal, and therefore the best test for the utility of remedies; and in order that he might effect his purpose of ascertaining the most efficient remedies impartially, neglecting the results of his own experience, derived from nearly 400 cases, he had investigated the numerous reports and returns to the government on the subject, and had thence selected those remedies which, from the statistics of the numbers cured by their use, or from the description of their direct effects, appeared to be most trustworthy; and he had the candour to state, that by this process he had been induced to regard with more favour certain remedies which a prejudice, derived from a limited experience, had led him to consider as worse than useless. In reply to those who thought no treatment of any service, he instanced the frightful mortality in India, Persia, and Russia, where little or no medical aid was procurable; and took occasion to congratulate the medical men of this country on the low rate of deaths (one-third) compared with the mortality in France or any country in Europe, which he attributed, under Providence, to their superior practical knowledge of disease in general. The idea of the nature of cholera that seemed most consistent with its symptoms and with the effects of treatment was, that it is the prolonged cold stages of a peculiar form of fever, presenting more analogies with continued than intermittent fever, the effect of a poisonous miasm, which, if very intense, destroys the patient, without the occurrence of any active symptoms, in a few hours; but in other cases induces a gradually increasing congestion of the internal venous system, which relieves itself by the effusion of serous fluid into the stomach and bowels, which is as speedily removed by the actions of vomiting and purging. The blood thus deprived of its serum becomes thick and black, and is deprived of its vital properties to such a degree, that, unable to imbibe oxygen in the lungs, it ceases to stimulate the heart, which organ is thus unable to propel the blood to the extremities or through the lungs. This is the stage of collapse, in which the patient is blue, cold, pulseless, and voiceless, and his features are shrunk to such a degree that his dearest and nearest relatives do not recognise him; and this is the most fatal stage and that least under the influence of remedies. Nevertheless, even under these deplorable circumstances we must not relax our exertions; and the list of curative means employed with success, proves that much may still be done for the relief of the patient. The most approved external remedies were, external heat, cold affusion, and counter-irritants. The author attempted to account for the favourable opinion of the application of cold in Persia, and of heat in Russia and the rest of the northern countries, partly by the concordance of such ideas with the ordinary feelings of the inhabitants of such different climates, and partly by the notion that in Persia, where the thermometer is above 98° in the sun, the patients, exhausted of their fluids by the disease, would be dried up and mummified by any attempt to keep up the temperature of the body by exposure to the sun's heat; whereas in Russia, &c., it was supposed, and probably with truth, that as cold is a direct sedative, its opposite, heat, was necessary to restore the vital powers. All the reports concur in the efficacy of cold affusion in inducing reaction; and its success appears to have been in proportion to the violence of the shock. Counter-irritants were successful only in connection with other remedies; but they deserve attention from their stimulating power, and their readiness of application. Bloodletting was useful in every stage in relieving the congestion and rousing the heart by removing the load that oppressed it. In the state of complete collapse salt-and-mustard emetics, to excite the system, followed by bleeding, was a favourable mode of treatment. The internal remedies most to be relied on in the stage of collapse may be classed under the heads of revulsives, stimulants, and specifics. The first class, besides emetics of salt and mustard, comprised tartar emetic and croton oil, and calomel in large doses. The effect of each was to check the vomiting and purging; but the three last were remarkable for their power of restoring the flow of bile, of a dark-green colour, when tartrate of antimony or croton oil had been used; and like blue ointment, when the calomel had been given. The action of all three is supposed by the author to be irritating to the mucous membrane of the stomach and bowels, of which it stops the secretion by changing the action of the part. The blood thus diverted from the membrane returns without loss into the portal system, and by the secretion of fresh bile, previously pent up in the gall-bladder, is expelled, the spasm of the duct having been relaxed by the irritation of its orifice, produced by the tartrate of antimony, calomel, or croton oil. The blue colour of the bile when calomel was used may perhaps be explained by the decomposition of the salt by the alkali of the bile. From the number of returns in its favour, besides the extensive experience of the author in its use, he is disposed to place most reliance on the croton oil, as its action is simply irritant, (many of the patients complaining that it made their throats sore when given in solution or suspension ;) whereas the tartar emetic is a direct sedative, and, hence, may be dangerous ; and the author had given calomel most extensively in large doses without ever having seen such effects produced as those which he has mentioned above from the reports of others. Stimulants, after a fair trial, were almost universally condemned ; and yet it is remarkable that M. Magendie had more success with his punch than any other of the Parisian physicians. His great rival Broussais was so unsuccessful, that he entirely relinquished his care of cholera patients at Val de Grace. Opium, either in small doses as a stimulant, or in large ones as a sedative, was equally unfit to be relied upon. The last class—specifics—comprises calomel, with or without opium, cold water, salines, and quinine, although the author never met with a single case of real cholera in which he could trace the recovery of the patient to the influence of calomel, nor ever observed that it produced any specific effect whatever ; still, from the almost unanimous approval it has met with, and his own experience of its benefit in English cholera, he would strongly recommend it for future experiment, as being, at the least, perfectly harmless, though taken in enormous doses. He would also adopt the use of cold water *ad libitum*, upon the faith of the reports in its favour, though his own experience is decidedly opposed to it. Salines, on the other hand, when well diluted, have, besides a number of most favourable reports of their efficacy, this hypothesis in their support—viz. that they restore to the blood by their endosmosis through the coats of the vessels, if not by their being absorbed directly into the circulation, the saline matter removed by the serous evacuations; whereas, the water, if it be not rejected by vomiting, as in the author's experience, could scarcely be absorbed, the tendency of the venous system being to empty itself; nor, if it were absorbed, could it supply to the blood those saline elements of which it had been deprived. In the author's experience, the best mode, though a painful one, of arresting vomiting in all cases, is to keep the stomach empty ; when, after a time, it will cease to suffer the action of vomiting, whatever that may be. From an attack of English cholera he suffered, and thus cured, the last efforts of which produced only bloody mucus, as well as from other similar results after emetics, the author believes that the stomach contracts itself during vomiting. Quinine, viewed in reference to the hypothesis of the intermittent nature of cholera, seems worthy of further trial than it has yet experienced. In conclusion, the treatment recommended by the author in the stage of collapse would be the following, and much in the same order as the remedies are stated; Cold affusions ; hot air ; external counter-irritation, and frictions ; venesection ; mustard-and-salt emetics ; cold water *ad libitum*, or Dr. Stevens' salines ; calomel, and tartrate of antimony, alternately, in large doses ; and, if all failed, croton oil.

Dr. Lankester spoke of the fallacies likely to arise from placing too much dependence on individual experience in this disease ; we could scarcely arrive at any definite conclusion from the treatment of fifty or a hundred cases in any single district. Every remedy which had been employed had its advocates, among others he might allude to one not mentioned by the author—carbonic acid, which was said to be most successful. We must, in the absence of sufficient information on the subject of treatment, reason from analogy, and after observing the symptoms, inquire what would relieve analogous symptoms in other diseases. Let them take the collapse, for instance; in this condition the blood all left the surface, and the internal organs were congested. To bring the circulation back to the skin was clearly indicated; hence the application of hot air to the surface, and stimulating liniments, were recommended and said to be used with success. In Russia, camphor liniments for this purpose have been found of great service. Another object was to supply to the blood the fluid which was lost by the copious watery evacuations. These evacuations took away the saline matters from the blood, and hence cold water with salines was no doubt an important agent. But salines, when so administered, must be given in small quantity in large quantities of water, for if the opposite were employed, it would defeat the object of the administration. The liver was evidently deranged in these cases, and it was most important to restore the action of this organ, for he believed it was quite proved that the presence of bile in the intestines was essential to the production of animal heat. Calomel was hence indicated, but not, he thought, in large doses; it might be combined with small doses of opium to prevent its being carried off by the bowels.

Dr. King had the charge of a large district during the prevalence of cholera. At first he was quite at sea how to act in the stage of collapse. He first employed Steven's plan of salines, with small doses of calomel and opium; but almost all the cases were fatal. He then pursued a stimulating plan, with a similar result. He ultimately adopted the system recommended and practised by Mr. French. He placed a pail of cold water by the bedside of each patient, and allowed them to drink *ad libitum;* when the patient began to vomit, he considered, as a general rule, that he would do well. He should mention that, in addition to the water, he gave large doses of calomel, which might have had a share in producing the result. Those who recovered were all more or less affected with the calomel. When hiccups came on, he considered the patient out of danger. He found bile in the evacuations after this treatment, the fæces being of the colour of the blue mercurial ointment. He did not think the heart the chief seat of the disease. All pregnant women died. He spoke of the necessity of perfect repose in the treatment of the disease, and stated his belief that many patients had died on removal to the hospital. More success attended those who were treated at their own homes. Croton oil, according to his experience, was not of service. Hot air applications did harm; cold air was preferable. Brandy and all stimulants were injurious.

Mr. French said, that in conducting the treatment of the collapse of cholera, before using indiscrimately the articles of the materia medica, we should study the curative process adopted by Nature to this end. We readily enough admit that this kind of knowledge is absolutely necessary in conducting the treatment of mechanical injuries, and that surgery is only a successful art when this principle is borne in mind. The mode in which reaction is accomplished by Nature from the state of collapse in cholera, is a process with which I am perfectly familiar, by direct observation, and it is very simple. It consists in absorption of water into the bloodvessels, and in vomiting. It may be confidently stated, that a patient who is no longer purged, and who is vomiting, is undergoing reaction in the most favourable way possible; just as "adhesion" constitutes the most favourable and rapid cure of certain wounds. But to state the method more fully, it may be thus expressed:—

1st. Absorption of water into the bloodvessels; the patient's intense thirst inducing him to take this fluid freely.

2dly. Nausea; which produces a general relaxation of the system, thus diminishing obstruction to the passage of the blood in the congested vessels.

3dly. Vomiting; which mechanically assists in driving forward the blood in the congested vessels.

In the more intense states of collapse, the process of reaction is not established before the vomiting has continued for three days. In slighter cases, collapse, reaction, and convalescence, may all occur in twenty-four hours. When, however, the disease pursues this course, cases which presented the most hopeless aspect may do perfectly well, either becoming directly convalescent, or if local congestions and inflammations ensue, they assume an active character, and are under the control of art, while if this process has been frustrated, they commonly pass into a state of congestive and typhoid fever, in which they either sink or recover with great difficulty. As to the essential nature of the disease, I believe it consists in a poisonous influence which is exerted directly on the heart, depressing its action; for these reasons:—

1st. The heart's action is always diminished in this disease.

2dly. No essential lesion of any other vital organs really exists.

3dly. All the other symptoms and physiological conditions admit of explanation on this hypothesis.

The first argument is admitted by all observers. It will be also admitted that the functions of the brain are remarkably well performed until the very period of death. Then, there is no asphyxia, for these reasons—the mechanical apparatus is undisturbed. We are familiar with the symptom, "coldness of the breath," (inconsistent with asphyxia;) the recumbent position, so necessary to the cholera patient, would not be suitable for the state of asphyxia. Dr. Parkes, who has recently published a work replete with interesting facts on cholera, denies the paralytic condition of the heart, because the left ventricle was always empty; he also found that the lungs were extremely collapsed, and infers that the cause of the arrest of circulation must be sought for in the blood itself. The average duration of the disease, in the cases in which Dr. Parkes made post-mortem examinations, was ten hours; the observations were made sometimes a quarter of an hour after death; in all the cases the heart was found inirritable to the stimulus of the knife, while the muscles contracted under this stimulus. With the hope of learning something on these points, I destroyed a rabbit in two hours by repeated doses of infusion of digitalis; the animal became gradually too feeble to move, breathed quickly, and died: on opening the body, the heart was still feebly pulsating. The lungs were completely collapsed, and the left lung was so firmly contracted, that it sank in water; it readily admitted of inflation. The right side of the heart contained blood, and there was a very small clot in the left auricle; but the left ventricle was perfectly empty. The heart was quite as irritable to the galvanic stimulus as the muscles. From this experiment I infer, that the influence of cholera on the circulation is exerted in the same way as that of digitalis. With regard to the condition of the blood in cholera, I believe it to be the best under the peculiar physiological condition of the patient. Blood in the normal state is intended to be constantly circulated with a regulated amount of force. If this force be exceeded, we find that the fluidity of the blood is increased, that it acquires a brighter hue, and that it possesses the power of coagulating more firmly when stagnant. The converse of all this obtains with diminished force of circulation. To this alteration in the constituents of the blood is possibly owing the absence of fibrinous deposits in the heart of the vessels; the retention of its homogeneous condition, and its adaptation for the re-admixture with water during reaction, so soon as the baneful effects of the poison shall cease to be exerted in the system. Thus, to sum up:—In proportion to the force with which the circulation is controlled, either death results, or the blood is diminished in quantity, and altered in quality, by a secretion from the alimentary canal—the overwhelming effects of sudden congestion and fibrinous deposits being probably thus prevented.

Dr. Ayres had found more success from the employment of stimulants, in the stage of collapse, than from any other remedies. He had given brandy in large quantities, and opium, with the view of relieving the cramps, which were so distressing. He differed from the views advanced by Mr. French, respecting the pathology of the disease, and thought that the loss of the fluid alone was sufficient to explain all the effects on the circulation.

Dr. Webster remarked that it was satisfactory to know from the Reports of the Registrar-General, that last week no case of cholera had occurred in London—that two had occurred in the first week of November and seven in October; and that in the summer quarter ending September 1847, there were only 98 deaths from this disease, whilst in the corresponding quarter of 1846, there were 197 deaths from this cause.

Dr. Ward, in reply, said that he confined his remarks in the paper to the treatment of the disease, more from the experience of others than from his own; but he might observe that, curiously enough, he was in a district close to that occupied by Dr. King, in Staffordshire, without knowing it, until the remarks made by that gentleman on the present occasion. He might observe that he had tried the cold water plan recommended by Mr. French. The first case was favourable to its employment, but future experience had shown him that it was not a successful plan of treatment. Pregnant women attacked with this disease after aborting, all died. He had employed croton oil in the fifteen cases, all of which recovered but one, who died from the secondary fever.—*London Med. Gaz.*

